# Clinical presentation and genotype-phenotype correlation of a novel pure duplication of 6p25.2–p22.3: a case report and literature review

**DOI:** 10.1186/s13039-026-00774-3

**Published:** 2026-06-18

**Authors:** Huamei Tang, Xiaoqing Xu, Wei Wu, Yuxue Wang, Yuying He, Junying Qiu, Tong Ou

**Affiliations:** 1 Medical Laboratory, Shenzhen Luohu People’s Hospital, Shenzhen, 518000 Guangdong China; 2 Prenatal Diagnostic Center, Shenzhen Luohu People’s Hospital, Shenzhen, 518000 Guangdong China; 3 Medical Laboratory Center, Shenzhen Luohu Hospital Group, Shenzhen, 518000 Guangdong China

**Keywords:** 6p duplication, Heart defects, Critical region, Potential modifier genes

## Abstract

Duplication of 6p is a rare genetic syndrome of which about 25% have one or more congenital cardiac defects including cardiac septal defects, pulmonary artery hypoplasia and patent ductus arteriosus. We present the first case of a fetus with functional single ventricle and persistent truncus arteriosus during prenatal diagnosis whose genomic analysis revealed a novel pure duplication of 6p25.2-p22.3. The duplication fragment was confirmed to be associated with intrachromosomal insertion from mother via chromosome karyotype. The presentation of this case aims to expand the existing knowledge regarding this rare condition and facilitate its diagnosis in the future. Based on the comparison of cases with 6p duplication syndrome, we notice that the 6p terminal region, especially at 6p25.1 to 6p25.2, could be the critical region associated with heart complications or anomalies of the pulmonary arteries. The duplication of the potential modifier genes, RIPK1 and FARS2, were proposed to be attributed to heart defects in our study and should be further researched.

**Supplementary Information:**

The online version contains supplementary material available at https://doi.org/10.1186/s13039-026-00774-3.

## Background

6p duplication syndrome is a rare pathogenic chromosomal abnormality with an incidence of less than one in a million. Possible mechanisms associated with the occurrence of 6p duplication are parental balanced translocation carriage, marker chromosome, tandem or inverted duplication, and interchromosomal insertion [1]. Until now, less than 50 cases have been reported worldwide and these cases have shown large differences of clinical features. The main symptoms of 6p duplication syndrome may involve craniofacial abnormalities, congenital heart defects, congenital anomalies of the kidney and urinary tract (CAKUT), ocular problems, brain malformations, growth retardation, developmental delay, intellectual disability and feeding problems [2]. In many cases, the duplication region is translocated to another chromosome and accompanied by deletions of segments in other chromosomes, which may contribute to the variation in phenotypes of 6p duplication syndrome [3–6].

Congenital heart disease (CHD) refers to structural abnormalities of the heart or major blood vessels present at birth [7]. It is the most common birth defect, impacting about 1% of all live-born infants [8]. Cardiac complications occur frequently in the syndrome, including atrial/ventricular septal defects, pulmonary stenosis, and patent ductus arteriosus [5, 9, 10]. The availability of chromosomal microarray testing as a standard clinical test has increased the awareness of the contribution of CNVs to congenital heart disease,. Clinical and research-based testing suggests that CNVs contribute to 10–15% of CHD [11]. As previously noted, current evidence suggests that the pathogenic contribution of CNVs to disease phenotypes may be substantially underestimated, owing to platform-specific detection biases in widely adopted technologies such as whole-exome sequencing (WES) and chromosomal microarray analysis (CMA) [12].

Gene(s) in the duplicated region of 6p may play a vital role in the development and function of the heart, but none of the dosage-sensitive candidate gene or genes has been elucidated. This case is the first to report a phenotypic presentation of functional single ventricle combined with truncus arteriosus. The present study’s purpose was to discuss the clinical and molecular cytogenetic findings of a fetus, who was followed up and diagnosed with functional single ventricle and pure partial trisomy 6p, which subsequently was found to be inherited from maternal translocation. Compared with the previous cases, we propose the minimum overlap region, 6p25.1-p25.2, as the crucial region and RIPK1, FARS2 as the potential modifier genes in the context of genotype-phenotype correlation.

## Case presentation

**Fig. 1:**
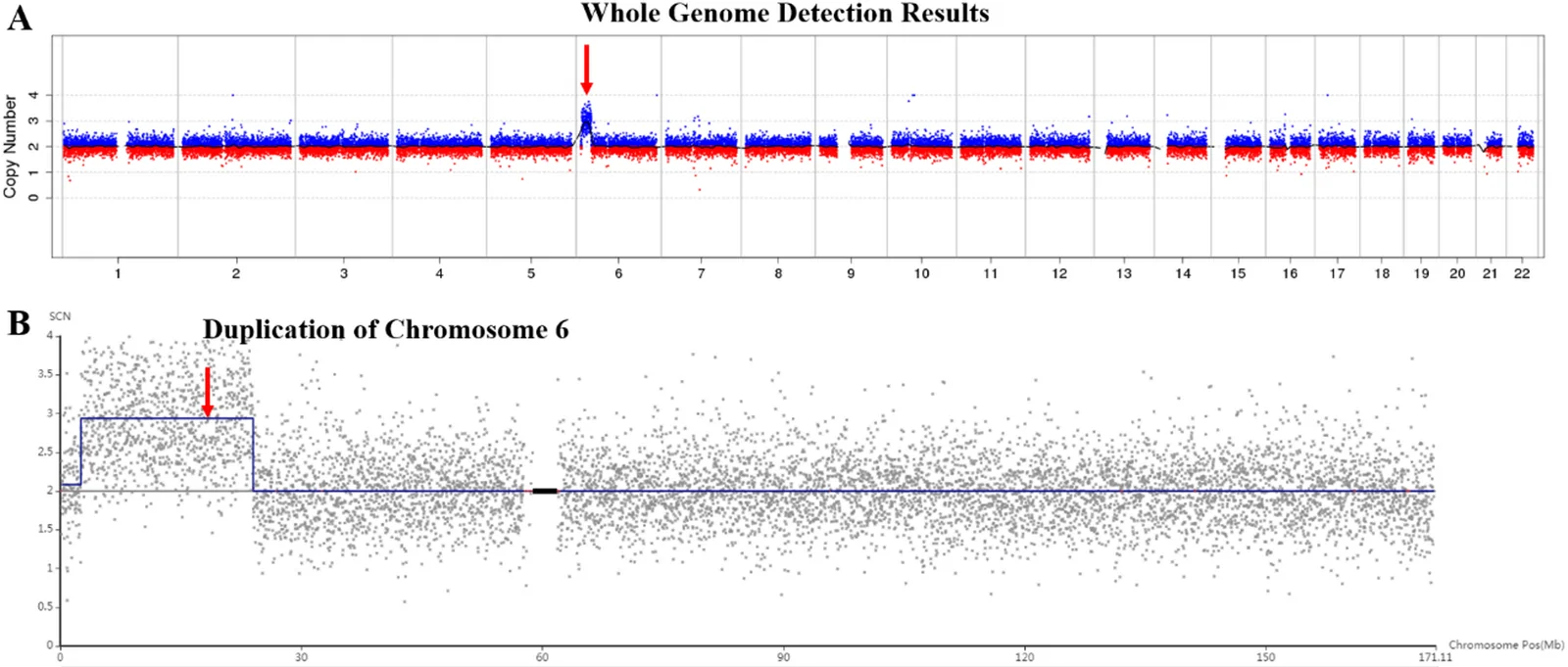
CNV-seq results of amniotic fluid. CNV-seq of amniotic fluid showed a 21.42-Mb duplication of chromosome 6p25.2–p22.3 (chr6:2580000–24000000) ×3. (A) Chromosomal view and (B) zoomed-in view.

**Fig. 2:**
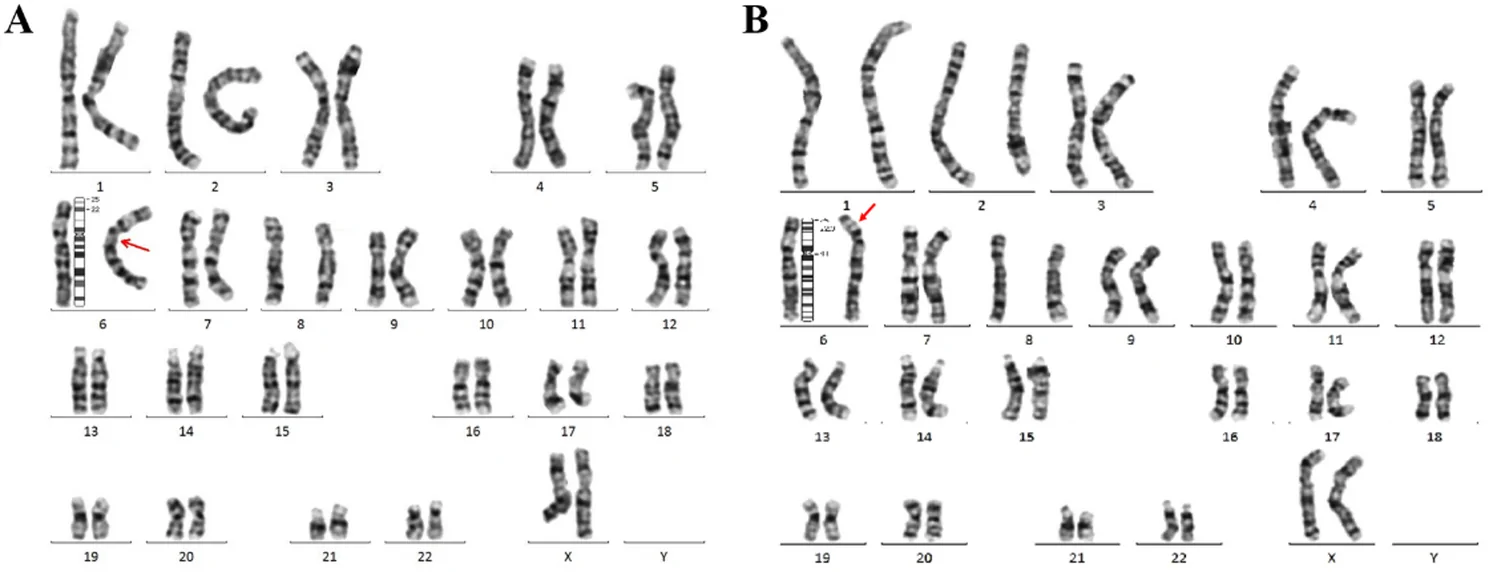
Karyotype analysis. (**A**) Cytogenetic analysis shows a 6p duplication in the fetus. (**B**) Karyotype shows a 6p insertion in the mother

**Fig. 3:**
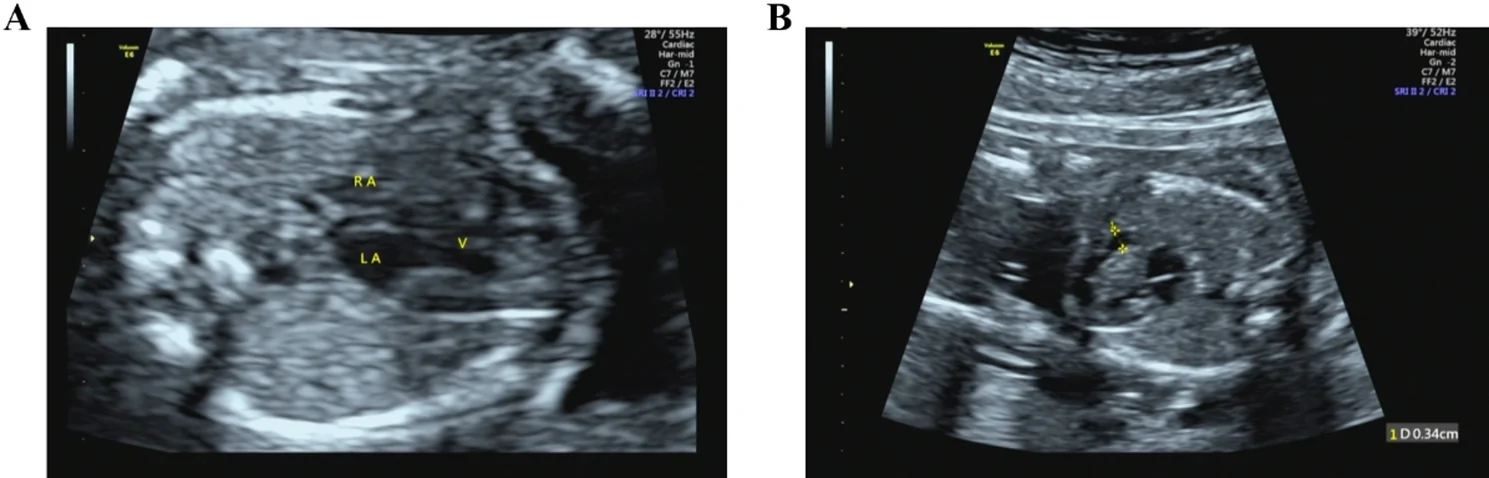
Fetal echocardiography at 19 weeks. (**A**) Functional single ventricle and atrioventricular valve (cardio-thoracic index<0.33). (**B**) The left and right ventricles were connected by a common arterial trunk and the effusion was seen in the pericardial cavity (3.4 mm)

A 28-year-old primigravida was referred to Prenatal Diagnostic Center of Luohu District People’s Hospital at 13 weeks of gestation. There were neither family history nor known diseases of both the pregnant woman and her spouse. The pregnancy was naturally conceived and not exposed to alcohol, irradiation, or infectious diseases. Prenatal ultrasound showed normal nuchal translucency thickness (1.8 mm) and the screening test for Down syndrome suggested low risk at 12 weeks. The result of NIPT indicated that 21-trisomy, 18-trisomy and 13-trisomy were negative, but showed a gain of approximately 22 Mb in the short arm of chromosome 6. Therefore, the woman accepted amniocentesis at 13 weeks of gestation after the consultation. Both conventional karyotyping and copy number variation sequencing (CNV-seq) were performed on amniocytes. The abnormal CNV-seq results revealed a 21.42-Mb duplication of chromosome 6p25.2-p22.3 (chr6:2580000_24000000) ×3[GRCh37/(hg19)] (Fig. 1). It was classified as a pathogenic CNV according to the ACMG (Medical Genetics and Genomics) guidelines. The results of amniotic fluid karyotyping analysis showed a 6p25.2-p22.3 duplication inserted into 6q11 (Fig. 2). The couple was also subjected to G-banding chromosomal analysis and CNV-seq analysis. No abnormalities were noted in the CNV-seq results of the parents. Karyotyping of the parental peripheral blood revealed 6p25.2-p22.3 inserted into 6q11 in the mother (Fig. 2) and the karyotype of the father was normal. The fetal echocardiography examination demonstrated a cardiac malformation at 19 weeks of gestation and the four-chamber view showed functional single ventricle, truncus arteriosus accompanied by pericardial effusion (Fig. 3). The parents decided to terminate the pregnancy. Our research was reviewed and approved by the Ethics Committee and Ethical Review Board.

## Materials and methods

### CNV-seq

Genomic DNA (40 ng) was fragmented and a DNA library was constructed according to the protocol. Multiple libraries were indexed, pooled, and then sequenced on the NextSeq CN500 instrument (Illumina, US). The CNVs were classified into five grades according to the American College of Medical Genetics and Genomics (ACMG) guidelines after CNV-seq testing and bioinformatics analysis.

### Chromosome karyotype analysis

For the prenatal sample, two cell cultures were set up by seeding the flasks with 10 mL of amniotic fluid, followed by trypsin-Giemsa banding. The amniotic culture was expanded for 8 days, and then G-banded (320–400 bands) karyotyping analyses were performed on metaphase cells according to standard protocols. The detected chromosomes were named according to the ISCN (International System for Human Cytogenetic Nomenclature).

### Short tandem repeats (STR) analysis

Genomic DNA (gDNA) was extracted from amniotic fluid (approximately 10 mL) by using a DNA Extraction Kit (Tissue and Cells) (QIAGEN, Hilden, Germany). Multiplex fluorescent PCR combined with 21 short tandem repeat (STR) markers was performed using the Microreader^TM^ 21 Direct ID System (Suzhou Yuewei Gene Technology Corporation, China) to measure MMC (maternal cell contamination) and identify polyploidy. The amniotic fluid sample had MCC levels less than 5% and qualified for CNV-seq analysis.

## Discussion and review of the literature

### 6p duplication syndrome

6p duplication syndrome can cause uncommon genetic disorders with varied manifestations from moderate to severe, depending on the size and area of the duplicated chromosome. The vast majority of the diagnoses are made during childhood, after karyotyping performed for neurodevelopmental disorders and/or intellectual disability. Several main symptoms of 6p duplication syndrome are reported, such as low birth weight, feeding problems and recurrent respiratory infections, growth and psychomotor retardation, several craniofacial abnormalities such as high forehead, close-set eyes with long eyelashes, ocular anomalies, high/wide nasal bridge, small mouth with thin lips, low-set and/or malformed ears [1]. Congenital heart and brain anomalies, as well as renal symptoms, seem to be variable [13].

In the vast majority of the cases, a paternal or maternal balanced translocation is responsible for the disorder when inherited, while *de novo* mutations appear rarely. The duplicated material from the 6p region may remain on the same chromosome or, more rarely can be translocated to a different one. Some of the 6p duplication syndrome cases reported in the literature include the partial monosomy of another chromosome, which makes it difficult to determine the genotype-phenotype relationship. The present study described a fetus diagnosed with pure partial trisomy 6p and followed up with functional single ventricle and common arterial trunk.

### Mechanisms of structural chromosomal rearrangement formation

Combining the CNV-seq and G-banding analysis findings of the fetus, we found a 21.42-Mb duplication of chromosome 6p25.2-p22.3 was inserted into 6q11, rather than a tandem duplication. While the CNV-seq and G-banding analysis of the primigravida showed the 6p25.2-p22.3 was translocated into 6q11. We speculated that the duplication of 6p25.2-p22.3 resulted from homologous recombination during meiosis. High-resolution analysis was available to determine the DNA sequence at, and near, the breakpoints in order to reveal how cells respond to repair broken ends [14].

### Correlation of our case and other cases reported

We compared the phenotypes of 6p duplication cases, which were reported from 1977 to 2025 (see supplementary Table 1). Among these features, heart abnormalities have been observed in about 51.11% of patients with 6p duplication syndrome. To date, among the 45 cases with 6p duplication syndrome, 23 patients have been reported to have one or more heart defects (Supplementary Table 1) [1, 5, 6, 9, 10, 15–22]. In terms of cardiac septal defects, the risk for ventricular septal defects (*n* = 6) or atrial septal defects (*n* = 8) was higher than others. In these cases, anomalies of pulmonary arteries were reported including pulmonary stenosis (*n* = 6), pulmonary hypertension (*n* = 2), bicuspid pulmonary valves (*n* = 1), and pulmonary artery atresia (*n* = 1). Therefore, we speculate that obstruction to pulmonary blood flow is one of the major characteristics of cardiac complications. Other common heart anomalies were also reported to be associated with distal 6p duplication, including patent ductus arteriosus (*n* = 5), patent foramen ovale (*n* = 4), heart murmur (*n* = 5), bicuspid aortic valve (*n* = 1) and Tetralogy of Fallot (*n* = 1). These findings were always accompanied by cardiac hypertrophy and pulmonary hypertension, which were probably secondary changes.

In our case, a fetus with functional single ventricle has not been reported previously, which is used to describe the congenital heart defect (CHD) with one functioning ventricle. Without multiple palliative surgeries after birth, the prognosis of these patients is poor and mortality is high. Establishment of a Fontan circuit is the standard treatment for patients with functional single ventricle, which separates the pulmonary and systemic circulations, but Fontan circulation can result in hemodynamic disturbances and a host of ensuing clinical complications with elevated systemic venous pressure and low cardiac output [23]. Although the prognosis for patients with functional single ventricle has improved, exercise intolerance, reduced the quality of life for adult survivors and life-threatening complications associated with multi-organ dysfunction are common [24].The persistent truncus arteriosus was also observed in prenatal diagnosis, which resulted from a failure of septation of the embryologic truncus or may occur as an isolated defect. Surgical repair is essential for these kinds of complex cardiac developmental anomalies, which contains separation of systemic and pulmonary circulation, as well as correction of any associated cardiac lesions. Even after surgery, the early mortality rate reached 5%, and the overall mortality rate was around 20% during a median follow-up period of approximately 12 years [25].

**Fig. 4:**
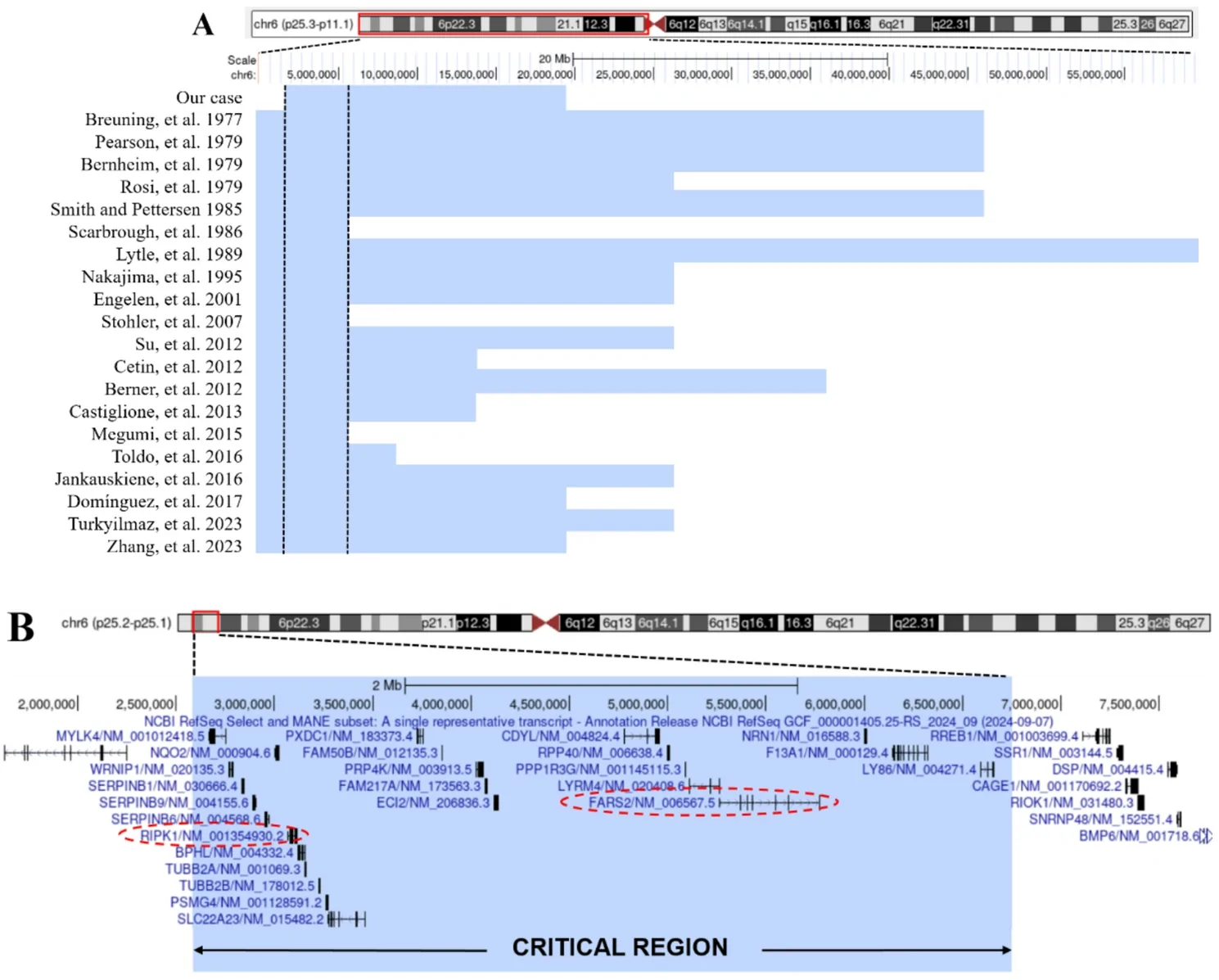
Schematic showing duplication involving 6p duplication. (**A**) Duplications of the previously reported patients with heart defects phenotype and congenital heart defects are depicted in light blue bars. The region of overlap amongst the duplications is 6p25.1 to 25.2, which maybe the critical region for heart defects. (**B**) There are many other potential modifier genes that are consistently duplicated in all these patients (within the critical region for congenital heart defects as depicted by the red circled region)

We compared the locations of cases with 6p duplication syndrome and noticed that the 6p terminal region, especially 6p25.1-6p25.2, could be the critical region associated with heart complications or anomalies of pulmonary arteries. We propose that the triplosensitivity of one or several genes located in the 6p25.1 to 6p25.2 region may contribute to the heart anomalies (Fig. 4).

### Gene associated with heart defects (genotype–phenotype correlation)

Abnormal heart development or congenital heart defects are complex polygenic disorders influenced by multiple genetic and environmental factors. Within the 6p25.1–p25.2 region, a total of seven OMIM morbid genes are located, including SERPINB6, RIPK1, TUBB2A, TUBB2B, FARS2, LYRM4, and F13A1. We reviewed the function and associated disease phenotypes of each gene from the OMIM database. SERPINB6 encodes a serine proteinase inhibitor 6, and it plays a critical role in the inner ear by protecting against the leakage of lysosomal contents during stress. The loss of this protective function leads to cell death and sensorineural hearing loss [26]. TUBB2A and TUBB2B encode tubulin and defects in these genes are associated with complex cortical dysplasia with brain malformations [27, 28]. Mutations in the LYRM4 gene, encoding the iron-sulfur cluster biogenesis factor ISD11, result in multiple respiratory chain complex deficiencies [29]. The F13A1 gene encodes the A subunit of factor XIII. The RIPK1 gene encodes a cytosolic protein kinase (Rip) that regulates various signaling pathways involved in inflammation and cell death through apoptosis or necroptosis [30]. Variation in RIPK1 is linked to autosomal dominant autoinflammation with episodic fever and lymphadenopathy (OMIM #618852) and autosomal recessive immunodeficiency 57 with autoinflammation (OMIM #618108) [30, 31]. Overexpression of Rip in mammalian cells induced morphologic changes characteristic of apoptosis [32], which may play an important role in pericardial effusion and ventricular septal defect. Moreover, according to their publicly available literature, the probability of triplosensitivity (pTriplo) for RIPK1 is 0.926, which is very close to the threshold (pTriplo > = 0.94) and is strongly predicted to be triplosensitive [33]. FARS2 encodes a protein that transfers phenylalanine to its cognate tRNA and plays a role in the mitochondrial translation machinery. Mitochondria are crucial for cardiac homeostasis, providing the essential energy for heart contraction and regulating key cellular survival and death processes [34]. Variation in FARS2 is linked to cardiac hypertrophy, left ventricular dilation or progressive heart failure by disrupting the mitochondrial quality control (MQC) system [35]. It is worth noting that FOXC1, a gene located at 6p25.3, plays an essential role in the development of cardiac outflow tract and pulmonary artery [36, 37]. However, FOXC1 was not included in the duplicated region in our case (6p25.2–p22.3). Therefore, its duplication cannot explain the cardiac phenotype observed in this fetus. Nevertheless, FOXC1 may play an important role in other 6p duplication cases with more telomeric breakpoints.

No other reported cases of 6p duplication syndrome with heart defects have specifically implicated these genes, and functional studies demonstrating a dosage‑sensitive mechanism leading to complex congenital heart defects remain limited. As a result, we propose RIPK1 and FARS2 as potential modifier genes that may act in concert with other dosage‑sensitive genes within the 6p25.2–p22.3 region. Nevertheless, more studies using animal models with targeted overexpression of RIPK1 or FARS2 in the developing heart are needed to test this hypothesis. Moreover, the possibility of cumulative effects of these genes on the fetal phenotype cannot be excluded. From a technical perspective, we performed CNV-seq (average resolution ~ 100 kb) and G-banded karyotyping on the fetal sample. We did not conduct higher-resolution sequencing, such as whole-exome sequencing (WES) or whole-genome sequencing (WGS), on this sample. Therefore, we cannot exclude the possibility that additional pathogenic single nucleotide variants (SNVs) or small insertions/deletions within the duplicated 6p25.2-p22.3 region may co-exist and contribute synergistically to the observed phenotype.

## Conclusions

We present a very rare case of a fetus with functional single ventricle and persistent truncus arteriosus during the prenatal diagnosis. Genomic analysis by CNV-seq revealed a pure partial trisomy of distal 6p, which was associated with an intrachromosomal insertion from the mother. The duplication of distal 6p chromosomal region could be associated with the development of heart complications in this syndrome. Review of the literature shows that heart malformations in the syndrome are various and a patient with functional single ventricle has not previously been reported. 6p25.1 to 6p25.2 would be the minimum crucial region that contributes to the heart anomalies. Furthermore, we hypothesize that RIPK1 and FARS2 may be potential modifier genes for investigating the association between 6p duplication syndrome and cardiac development. Further studies are necessary to elucidate the contributions of these genomic regions and genes in individual cardiac development and to explore their associated polymorphisms. The combination of several approaches, including fetal ultrasound techniques, karyotype analysis and CNV-Seq, could provide important insights into difficult prenatal diagnoses, which is helpful for early management and genetic counseling.

## Electronic Supplementary Material

Below is the link to the electronic supplementary material.

Additional file 1: Table S1.

## Data Availability

No datasets were generated or analysed during the current study.
