# Supplementary material for: Clinical presentation and genotype-phenotype correlation of a novel pure duplication of 6p25.2–p22.3: a case report and literature review
Source: Mol Cytogenet. 2026 Jun 18;19:32. doi: 10.1186/s13039-026-00774-3 (PMC13543468; doi:10.1186/s13039-026-00774-3)
Supplement: Supplementary file 1 [file 13039_2026_774_MOESM1_ESM.docx]

Supplementary Table 1 Comparison of cases with 6p duplication syndrome (from 1977 to 2025)

| number | Author | Location | Congenital heart defects | Phenotypic features |
| --- | --- | --- | --- | --- |
| 1 | Chiyo, H, et al. 1975[38] | 6pter -6p21 | **-** | Mental retardation, prominent forehead, blepharoptosis, epicanthic folds, strabismus, low-set ears, bulbous nose and sacral dimple. |
| 2 | Breuning MH, Bijlsma JB, France HFD.1977[6] | 6pter -6p21 | **+** | Low birth weight, psychomotor retardation, craniofacial abnormalities (such as high prominent forehead, large fontanel, wide sagittal suture, blepharoptosis, low-set and/or malformed ears), congenital heart malformation(ventricular septum defect and hypertrophy of the heart, bicuspid aortic and pulmonary valves; two-lobed right lung; mobile colon ascendens; the mesocolon connected with the mesenterium), small kidneys, and proteinuria. |
| 3 | Cote GB, Papadakou-Lagoyanni S, Sbyrakis S. 1978[39] | 6pter to 6p23 and part of 6p22 | **-** | Low birth weight and height, psychomotor retardation, feeding problems, micrognathia, cataracts, proteinuria, odd facies, omphalocele, no heart defect could be detected. |
| 4 | Pearson G, et al. 1979[40] | 6pter -6p21 | **+** | Facial dysmorphia, cranial synostosis, micro-opthalmia and blepharophimosis, multiple hemangiomas of skin, talipes equinovarus, persistent patent ductus arteriosis, absence of uterus and upper vagina, polymicrogyri, and arhinencephaly. |
| 5 | Bernheim A, et al. 1979[15] | 6pter -6p21 | **+** | Psychomotor retardation, blepharoptosis, high nasal bridge, nystagmus, small mouth, systolic murmur showed signs of ventricular hypertrophy. |
| 6 | Rosi G, et al. 1979[16] | 6pter -6p22 | **+** | Low birth weight, feeding problems, psychomotor retardation, blepharoptosis, micrognathia, congenital cardiopathy, heart murmur, interventricular defect of moderate severity. |
| 7 | Ferrando P, et al. 1981[41] | 6pter -6p22.4 | **-** | Low birth weight, psychomotor retardation, low-set ears, blepharoptosis, high forehead, clinodactyly. |
| 8 | Eden MS, et al. 1985[42] | 6pter -6p21 | **-** | Low birth weight, psychomotor retardation, microcephaly, blepharoptosis, , high myopia with blepharospasms and ptosis, mixed hearing loss. |
| 9 | Smith BS, Pettersen JC. 1985[17] | 6pter -6p21 | **+** | Patient 1: Ventricular septal defect, pulmonic stenosis with mild left to right shunt.  Patient 2: The heart had an asymmetrical appearance owing to a rather large left ventricle and a comparatively small right ventricle. The right ventricle had a very thick wall and bulged into the left ventricular chamber. The pulmonary trunk was completely atretic at its base. |
| 10 | Scarbrough PR, et al. 1986[18] | 6pter-25 | **+** | Microcephaly, blepharoptosis, mild hypertelorism, holosystolic murmur and/or defect, syndactyly |
| 11 | Burd L, et al. 1988[43] | 6pter -6p23 | **-** | Low birth weight, psychomotor retardation, prominent forehead, low-set ears, prominent nasal bridge |
| 12 | Lytle C, et al. 1989[10] | 6pter -6p11 | **+** | Low birth weight, microcephaly, low-set ears, pulmonary stenosis, ventriculoseptal defect, patent ductus arteriosus |
| 13 | Nakajima W, et al. 1995[9] | 6pter -6p22 | **+** | Low birth weight, microcephaly, broad forehead, blepharoptosis, small mouth, ultrasound cardiogram showed small atrial septal defect |
| 14 | Rudnik-Schoneborn S, et al. 1997[44] | 6pter -6p21 | **-** | Low birth weight, psychomotor retardation, small mouth, low-set ears, muscular hypotonia，no evidence of congenital heart defect or of renal hypoplasia. |
| 15 | Röthlisberger B, et al. 1999[45] | 6pter -6p23 | **-** | Low birth weight, psychomotor retardation, microcephaly, blepharoptosis, abnormal ears |
| 16 | Villa A, et al. 2000[46] | 6p21.3-6p12 | - | Low birth weight, psychomotor retardation, seizures, microcephaly, narrow forehead, low-ser ears |
| 17 | Engelen JJ, et al. 2001[5] | 6pter-6p22.1 | **+** | Low birth weight, a dilated and severely hypertrophic right ventricle and an atrial septal defect II. supersystemic pulmonary hypertension with multiple peripheral pulmonary stenoses and a patent ductus arteriosus with right to left shunting, growth retardation, Facial anomalies, a left sided hydronephrosis and a grade IV±V vesico ureteral reflux, |
| 18 | Ng D, et al. 2001[47] | 6p22.2-6p21.1 | **-** | Low birth weight, psychomotor retardation, prominent forehead, microstomia, low-set ears. |
| 19 | Giardino D, et al. 2002[48] | 6pter -6p22 | **-** | Low birth weight, psychomotor retardation, hypertrophy, strabismus, proteinuria, reduced number of glomeruli |
| 20 | Dominguez MG, et al. 2003[49] | 6p23-6p21.2 | **-** | Low birth weight, hypotrophy, microcephaly, prominent forehead, blepharoptosis, small mouth, low-set ears. |
| 21 | Vermeesch JR, Thoelen R, Fryns JP. 2004[50] | 6pter -6p24.1 | **-** | psychomotor retardation, severe speech developmental delay and mild dysmorphic features. |
| 22 | Fogu G, et al. 2007[51] | 6p12.1-6p22.1 | **-** | craniofacial dysmorphisms, anal atresia, hypotonia. |
| 23 | Stohler R, et al. 2007[52] | 6pter -6p25.1 | **+** | dysmorphic features, respiratory distress, episodes of cyanosis, hearing loss, atrial septal defect with left to right shunting and mild peripheral branch pulmonary stenosis and a renal ultrasound showed left-sided, grade II hydronephrosis. |
| 24 | Andrieux J, et al. 2008 [53] | 6p22.3-6p21.2 | **-** | Low birth weight, psychomotor retardation, epilepsy, epicanthus, hypertelorism |
| 25 | Bart IY, et al. 2011[54] | 6p21.31-6p12.2 | **-** | Psychomotor retardation, tracheomalacia, micrognathia, macroglossia, feeding problems |
| 26 | Su PH, et al. 2012[55] | 6pter -6p22 | **+** | craniofacial anomalies, persistent hyperplastic primary vitreous with retinal detachment. Feeble movements, general hypotonia, and recurrent vomiting were noted. patent foramen ovale with left to right shunt. |
| 27 | Cetin Z, et al. 2012[56] | 6pter -6p23 | **+** | craniofacial anomalies, hydrocephaly, colpocephaly, bilateral ventricular dilatation and hypogenesis of corpus callosum, ventricular septal defect and secundum atrial septal defect. |
| 28 | Berner AL et al. 2012[57] | 6p25–6p21.3 | **+** | Low birth weight, psychomotor retardation, blepharoptosis, proteinuria, small atrial septal defect (secundum type) |
| 29 | Varvagiannis K, et al. 2013[58] | 6p21.1-6p12.3 | **-** | Psychomotor retardation, prominent forehead, micrognathia, strabismus, scoliosis |
| 30 | Castiglione A, et al. 2013[19] | 6pter-6p23 | **+** | Psychomotor retardation, hearing loss, heart defects, renal dysfunction,  First born: patent foramen ovale, patent ductus arteriosus; 1–2/6-grade mesocardic heart murmur  Second born: 1–2/6-grade mesocardic heart murmur. |
| 31 | Savarese M, et al. 2013[59] | 6p21.31-6p12.3 | **-** | mild dysmorphic features, moderate intellectual disability with behavioral disturbances, immunodeficiency, and epilepsy. ECG, and echocardiography were unremarkable. |
| 32 | Chen CP, et al. 2014[60] | 6pter-6p22.3 | **-** | craniosynostosis, closed anterior fontanelles, prominent forehead, microcephaly, short bulbous nose, long philtrum, small mouth, micrognathia, low-set ears and clinodactyly |
| 33 | Yoshimura-Furuhata M, et al. 2015[13] | 6pter-p25.1 | **+** | Psychomotor retardation, hydronephrosis, proteinuria, glomerular sclerosis, hypotonia and sucked poorly, hyperopia, ventricular septal defects, patent ductus arteriosus, and tetralogy of Fallot. |
| 34 | Toldo I, et al. 2016[21] | 6pter-6p24.3 | **+** | congenital heart defect, mild facial dysmorphism, mild neurodevelopmentaldelay and borderline cognitive profile. |
| 35 | Jankauskien A, et al. 2016[20] | 6pter-6p22.1 | **+** | Low birth weight, psychomotor retardation, hearing loss, hypotonia, hydrocephalus, microstomia, pulmonary stenosis, proteinuria |
| 36 | Sivasankaran A, et al. 2017[61] | 6pter-6p22.3 | **-** | Low birth weight, psychomotor retardation, low-set ears, macrocephaly |
| 37 | Saori M, Masanori T, Takeshi Y. 2017[22] | unknown | **+** | Short stature, psychomotor retardation, pulmonary stenosis, atrial septal defect |
| 38 | Domínguez MG, et al. 2017[62] | 6pter-6p22.3 | **+** | Weak suck, cyanosis, microphthalmia. patent ductus arteriosus and foramen ovale, mild pulmonary valve stenosis, mild right ventricular hypertrophy, and bilateral renal hypoplasia. |
| 39 | Peterman LA, et al. 2020[4] | 6pter-6p24.3 |  | Psychomotor retardation, seizures, microcephaly, hydrocephalus, corpus callosum agenesis, scoliosis |
| 40 | Turkyilmaz A, et al. 2023[1] | 6pter-6p22.1 | **+** | Low birth weight, growth retardation, prominent forehead, short and narrow palpebral fissures, blepharoptosis, convex nasal ridge, hemangioma on the left eyelid, high-arched palate, retromicrognathia, and low-set ears, hearing loss, minimal dilatation of the left kidney renal pelvis, atrial septal defect |
| 41 | Zhang L, et al. 2023[3] | 6pter-6p22.3 | **+** | abnormal growth, developmental delay, skeletal dysplasia, hearing loss, and dysmorphic facial features. patent ductus arteriosus, atrial septal defect, and pulmonary hypertension. congenital heart defects, such as patent foramen ovale, coronary sinus dilation, and persistent left superior chamber. |
| 42 | Yu Y, et al. 2024[63] | 6pter-6p22.3 | **-** | unexplained growth and intellectual development delay, multiple craniofacial malformations, and recurrent infections of the upper respiratory tract. |
